# Vitamin D metabolism in critically ill patients with acute kidney injury: a prospective observational study

**DOI:** 10.1186/s13054-024-04869-4

**Published:** 2024-04-02

**Authors:** Lynda K. Cameron, Lesedi Ledwaba-Chapman, Kieran Voong, Geeta Hampson, Lui G. Forni, Nina Seylanova, Dominic J. Harrington, Rosario Lim, Aneta Bociek, Wang Yanzhong, Marlies Ostermann

**Affiliations:** 1 Department of Critical Care, King’s College London, Guy’s and St Thomas’ NHS Foundation Trust, London, SE1 7EH UK; 2 Pharmacy Department, Guy’s and St Thomas’ NHS Foundation Trust, London, UK; 3 Institute of Pharmaceutical Sciences, School of Cancer and Pharmacy, King’s College London, London, SE1 9RT UK; 4 Department of Population Health Sciences, King’s College London, Addison House, Guy’s Campus, London, SE1 1UL UK; 5 Nutristasis Unit, Synnovis, Guy’s and St. Thomas’ NHS Foundation Trust, London, UK; 6 Department of Chemical Pathology and Metabolic Medicine, St Thomas’ Hospital, Lambeth Palace Road, London, SE1 7EH UK; 7 Department of Diabetes and Endocrinology, Metabolic Bone Clinic, St Thomas’ Hospital, London, UK; 8 Department of Critical Care, Royal Surrey Foundation Trust, Guildford, Surrey UK; 9 School of Medicine, University of Surrey, Guildford, Surrey UK

**Keywords:** Acute kidney injury, Vitamin D, Colecalciferol, Parathyroid hormone, AKI

## Abstract

**Background:**

Vitamin D deficiency in critically ill patients is associated with poor outcomes, and vitamin D supplementation is recommended for patients with chronic kidney disease. Whether acute kidney injury (AKI) is associated with altered Vitamin D metabolism is unknown. We aimed to compare the longitudinal profiles of serum 25(OH)D and 1,25(OH)_2_D concentrations in critically ill patients with and without moderate to severe AKI and explore the impact of renal recovery and parathyroid hormone (PTH).

**Methods:**

In this prospective, observational study in two centres in the UK, critically ill patients with and without AKI underwent serial measurement of serum 25(OH)D and 1,25(OH)_2_D and plasma PTH concentrations for 5 days. Linear mixed model analysis and sensitivity analyses were performed.

**Results:**

Serial data of 137 patients were analysed. Seventy-one patients had AKI stage II/III of whom 23 recovered kidney function during the 5-day study period; 66 patients did not have AKI at enrolment of whom 14 developed new AKI. On day of enrolment, patients’ serum 25(OH)D concentrations were low (median 18 nmol/L) but there was no significant difference between patients with and without AKI. Median serum 1,25(OH)_2_D levels were significantly lower in patients with AKI II/III (41 pmol/L [IQR 26, 58]) compared to similarly unwell patients without AKI (54 pmol/L [IQR 33, 69]) during the 5-day period. Recovery of kidney function in patients with AKI was associated with a rise in 1,25(OH)_2_D concentrations. Plasma PTH results were impacted by serum calcium and magnesium levels but not associated with 1,25(OH)_2_D levels.

**Conclusions:**

Critically ill patients with moderate-to-severe AKI have significantly lower serum 1,25(OH)_2_D concentrations than similarly sick patients without AKI but there was no difference in serum 25(OH)D concentrations. Recovery of AKI was associated with a rise in serum 1,25(OH)_2_D concentrations. More research is needed to investigate the health benefits and safety of supplementation with active vitamin D in critically ill patients with moderate-to-severe AKI.

*Trial registration* Clinicaltrials.gov (NCT02869919), registered on 16 May 2016.

**Supplementary Information:**

The online version contains supplementary material available at 10.1186/s13054-024-04869-4.

## Background

Vitamin D deficiency affects about 40% of Europeans. [1] It is associated with adverse health outcomes, including increased mortality, muscle weakness and osteoporosis, increased susceptibility to sepsis, longer duration of mechanical ventilation and increased risk of acute kidney injury (AKI). [2]

In health, more than 95% of systemic vitamin D3 (Vit D3), also known as colecalciferol, is synthesized in the skin under ultraviolet B (UVB) irradiation and then converted to 25-hydroxyvitamin D (25(OH)D) by 25-hydroxylase in the liver, followed by hydroxylation to 1,25(OH)_2_D (also known as calcitriol) predominantly in proximal tubular cells. [3] The production of 1,25(OH)_2_D is tightly controlled, being stimulated by parathyroid hormone (PTH), and inhibited by calcium, phosphate and fibroblast growth factor-23 (FGF-23). [3] 1,25(OH)_2_D, the main bioactive metabolite of vitamin D, exerts the greatest physiological effect.

In chronic kidney disease (CKD), 1,25(OH)_2_D levels start to decline when glomerular filtration rate (GFR) falls to < 60 ml/min. To promote bone and cardiovascular health in patients, routine vitamin D supplementation (using alfacalcidol) is recommended for patients with CKD. [4] Acute kidney injury is a syndrome with dynamic trajectories. [5] Whether there is also a role for routine Vitamin D supplementation in patients with AKI is unknown. [6] A systematic review of data of 130 critically ill patients showed no difference in serum 25(OH)D levels between patients with and without AKI but significantly lower serum 1,25(OH)_2_D levels in AKI patients. [7] A population-based cohort study from Taiwan showed that patients with AKI who had dialysis and survived 90 days after hospital discharge had a significantly higher long-term risk of bone fractures compared to hospitalized patients without AKI, regardless of subsequent progression to end-stage kidney disease (ESKD). [8] The authors speculated that changes in vitamin D metabolism might have contributed but did not measure vitamin D levels.

Finally, several randomized controlled trials (RCTs) have investigated the role of vitamin D supplementation in critically ill patients, with and without AKI. A meta-analysis of 14 trials involving 2324 patients concluded that the supplementation of vitamin D had no effect on overall mortality. [9] Importantly, in 12 of 14 trials, Vitamin D3 (colecalciferol) was used for supplementation. Whether other active Vitamin D or other vitamin D metabolites would have been more effective is unclear. A more recent meta-analysis of 16 RCTs including 2449 patients also showed that different types of Vitamin D had been used in clinical trials. [10] Further, in eight studies, vitamin D was administered via the enteral route, whereas in seven studies, Vitamin D was administered intravenously or intramuscularly. The VITDALIZE is still actively recruiting. [11] In this international placebo-controlled double-blind RCT, 2400 adult patients with severe vitamin D deficiency are randomized to receiving a loading dose of 540,000 IU cholecalciferol, followed by 4000 IU daily for 90 days versus placebo.

The main objectives of this study were (i) to undertake serial measurements of 25(OH)D and 1,25(OH)_2_D concentrations in critically ill patients with and without moderate to severe AKI; (ii) to investigate whether there are differences in vitamin D metabolite concentrations between AKI patients who recover renal function and those without recovery; and (iii) to investigate the role of factors that usually impact vitamin D regulation, including gender, annual season, ionized calcium (iCa), Magnesium (Mg) and PTH [12].

## Methods

### Setting

This prospective observational study was conducted in two tertiary care hospitals in the National Health Service in the UK. Patients were recruited between July 2016 and January 2018. Data collection and laboratory analyses were completed in the following 18 months. Statistical analyses were delayed due to the COVID pandemic but completed in 2022.

### Definitions

AKI was defined according to the serum creatinine and urine output criteria of the Kidney Disease Improving Global Outcomes (KDIGO) classification. [13, 14] Baseline kidney function was determined using the lowest serum creatinine result recorded in the patients’ medical notes in the 12 months before hospitalization. If no result was recorded in the medical notes, the primary care clinician and/or local hospital was contacted for additional information. If no baseline creatinine value was available, and there was no documented diagnosis or clinical suspicion of CKD, it was assumed that the patient’s baseline estimated glomerular filtration rate (eGFR) was > 45 mL/min/1.73 m^2^, and the serum creatinine was back calculated using the Modification of Diet in Renal Disease formula. [14]

Depending on the trajectories of kidney function during the 5-day follow-up period, we distinguished between patients with ‘persistent AKI II/III’, ‘no AKI throughout’, ‘development of new AKI’ and ‘AKI with recovery of kidney function’ during the 5-day period. [15] Kidney recovery was defined as a reduction in AKI stage or return to baseline kidney function, as per 16 th Acute Disease Quality Initiative expert consensus meeting. [15]

### Participants

Consecutive adult patients in the intensive care unit (ICU) were screened daily for eligibility. [12] Patients were included in the AKI group if they were critically ill adults and had KDIGO defined AKI stage II or III for 36 h or less. Criteria for inclusion in the critically ill non-AKI group were: (i) no AKI, and (ii) concomitant cardiovascular and/or respiratory failure necessitating invasive or non-invasive respiratory support and/or treatment with catecholamines, with a requirement anticipated to last for longer than 24 h. Criteria for exclusion from either group were: (i) AKI stage I; (ii) AKI stage II or III for more than 36 h; (iii) known CKD stage 3b–5 (i.e. baseline eGFR < 45 mL/min/1.73m^2^); (iv) previous renal transplant; (v) known vitamin D deficiency (a documented diagnosis or laboratory Vitamin D value of < 50 nmol/L); (vi) known vitamin D supplementation in the last 3 months (either as a single agent or in a combination product); (vii) known hyperparathyroidism; (viii) need for total parenteral nutrition (TPN); (ix) life expectancy less than 48 h; (x) haemoglobin concentration < 70 g/L, or (xi) pregnancy. These exclusion criteria were agreed in order to eliminate any factors that may confound serum vitamin D metabolite concentrations. If patients were included but developed exclusion criteria during the 5-day follow-up period (i.e. initiation of TPN or vitamin D administration for clinical reasons), they were excluded from the analysis and replaced with another patient.

### Study procedures

Blood samples for serial measurement of serum 25(OH)D and 1,25(OH)_2_D and plasma PTH were taken in the morning between 08.00 and 12.00 noon on the day of enrolment (within 36 h of the diagnosis of AKI stage II or III) and on day 5. Additional blood samples were taken on day 2 for measurement of serum 1,25(OH)_2_D concentration, owing to its shorter elimination half-life (~ 4 h). All samples were centrifuged at 3500 rpm for 10 min, then separated into aliquots and stored at − 80 °C until batch analysis after completion of patient recruitment.

### Data collection

The following data were collected from the medical records: baseline demographics, type of organ failure, type of organ support, severity of illness [Acute Physiology and Chronic Health Evaluation (APACHE) II and Sequential Organ Failure Assessment (SOFA) score and SOFA minus renal score (SOFA-Renal)], routine biochemistry results and C-reactive protein (CRP), and ICU and hospital mortality.

### Laboratory analysis

Serum concentrations of 25(OH)D and 1,25(OH)_2_D were measured using a liquid chromatography–mass spectrometry method, with a coefficient of variation (CV) of 7.3% for 25(OH)D. The lower limit of detection was 7 nmol/L. 1,25(OH)_2_D was measured using a standard ChemiLumunescent ImmunoAssay (Diasorin Liaison®). The intra-assay CVs were 1.9–2.3% across a range of concentrations from 39 to 200 pmol/L. The lower limit of detection was 12 pmol/L. Plasma PTH was measured by Roche automated analyser (Roche diagnostics Limited, West Sussex, UK). The reference range was 10–65 ng/L. Assay CVs for PTH were < 5% at PTH concentrations of 41 and 105 ng/L.

### Sample size

The aim of this study was to investigate Vitamin D physiology in patients with and without AKI during a 5-day period. Based on data from Lai et al. [16], a sample size of 126 patients with complete data was considered to be necessary to detect a difference of 25 pmol/L in serum 1,25(OH)_2_D concentration between the AKI and non-AKU cohort, with a power of 80% and at a two-tailed significance level of 5% (assuming a standard deviation of 50 in each group).

To study trajectories in Vitamin D concentrations, it was important to have blood results available on day 5 for all enrolled patients. After 50 patients had been recruited, we performed an interim review of the case report forms and noted that only 28 patients (58%) were alive and still in the ICU and had blood samples taken on day 5. Based on these results, we concluded that we needed to recruit at least 230 patients in order to be able to analyse 126 patients with day 5 samples. To compensate for potential missing or incomplete data, we aimed to recruit at least 136 patients.

### Statistical analysis

For the baseline characteristics, continuous variables were summarized using medians, and interquartile ranges (IQRs) and categorical variables were summarized using counts and percentages. Sequential Vitamin D metabolite concentrations were compared within the AKI and no AKI groups using the Mann–Whitney *U* Test. Multiple comparisons were accounted for by adjusting p-values with the Benjamini–Hochberg procedure.

For the inferential analysis, we used a linear mixed model which accounted for lack of independence of data within patients by modelling both fixed and random effects. We defined time as the number of days since enrolment. Model parameters were estimated using restricted maximum likelihood. In each model, we adjusted for kidney function, time, age, gender, ethnicity, season, and baseline CRP, ionized calcium concentrations, and ‘SOFA—Renal’ score. One model includes an interaction between kidney function (no AKI, persistent AKI II/III, new AKI, recovery of kidney function following AKI II/III) and time. We considered three different random effect structures, random intercept, random intercept with an uncorrelated slope, and random intercept with a correlated slope. We explored whether allowing the residual variances of each time point and/or kidney function improved the model. Satterthwaite’s method was used to estimate the p-values and confidence intervals (CIs) of the fixed effects. The model with the smallest Bayesian information criterion was chosen as the final model. We defined statistical significance at *p* < 0.05. All analyses were performed using R v4.1.3 and mixed models were fit and tested using the lme4 and lmerTest packages, respectively. As a sensitivity analysis, we took the natural logarithm of the outcome before fitting the model to see how stable the model coefficients were.

### Trial registration

The study was registered with clinicaltrials.gov (NCT02869919) on 16 May 2016.

## Results

### Baseline characteristics

**Fig. 1:**
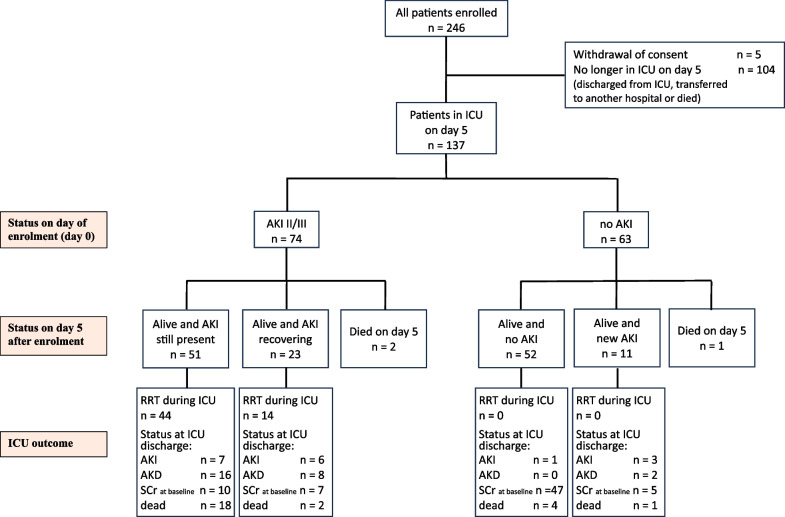
Flowchart *AKI* Acute kidney injury, *AKD* Acute kidney disease, *ICU* Intensive care unit, *RRT* Renal replacement therapy, *SCr* at baseline Serum creatinine returned to baseline. * patient developed new AKI after day 0 but died on day 5

Patient recruitment ceased after enrolment of 137 patients with complete data for analysis (Fig. 1, Table 1). Seventy-four patients had AKI stage II/III of whom 59 were treated with renal replacement therapy (RRT); 23 patients recovered kidney function during the 5-day follow-up period. Sixty-three patients did not have AKI at enrolment of whom 11 developed new AKI during the 5-day study period. (Fig. 1) Patients with AKI II/III at enrolment had significantly higher APACHE II and ‘SOFA–Renal’ scores at ICU admission compared to patients without AKI at enrolment but there was no statistically significant difference in age and gender. (Table 2) ICU, hospital and 30-day mortality were significantly higher in the AKI II/III cohort.

**Table 1:** Baseline characteristics, organ support and outcomes

Variable	Category	All	No AKI at enrolment	AKI II or III at enrolment	p	Missing data (%)
N		137	63	74		
Age, in years		54 [47, 68]	52 [46, 69]	57 [47, 68]	0.410	0.7
Gender	Male	91 (66)	40 (63)	51 (69)	0.587	–
	Female	46 (34)	23 (37)	23 (31)		
Ethnicity	Asian	4 (3)	3 (5)	1 (1)	0.273	3.6
	Black	9 (7)	2 (3)	7 (9)		
	Caucasian	118 (86)	54 (86)	64 (86)		
	Other	1 (1)	1 (2)	0 (0)		
	Missing	5 (4)	3 (5)	2 (3)		
Body weight at admission to ICU	Body weight [kg], mean	81.9 (21.4)	80.6 (23.8)	83.1 (19.2)		–
	Body mass index	27.2 (6.7)	26.4 (7.8)	27.8 (5.7)		–
Admission diagnosis	Medical	95 (69)	45 (71)	50 (68)	0.585	1.5
	Surgical	40 (29)	18 (29)	22 (30)		
	Missing	2 (1)	0 (0)	2 (3)		
Sepsis at admission to ICU	Yes	85 (62)	38 (60)	47 (64)	0.727	–
Sepsis at enrolment	Yes	83 (60.6)	36 (57,1)	47 (64)	0.486	–
	Missing	2 (1)	0 (0)	2 (3)		
Chronic comorbidities	Chronic lung disease	37 (27)	21 (33)	16 (22)	0.176	–
	Chronic heart failure	15 (11)	8 (13)	7 (9)	0.592	–
	Coronary artery disease	30 (22)	11 (17)	19 (26)	0.302	–
	Chronic liver disease	13 (9)	3 (5)	10 (14)	0.141	–
	CKD 1 – 3a	6 (4)	2 (3)	4 (5)	0.687	–
	Chronic GI disease	15 (11)	8 (13)	7 (9)	0.592	–
	Diabetes	20 (15)	5 (8)	15 (20)	0.053	–
	Hypertension	50 (36)	21 (33)	29 (39)	0.593	–
	Cancer	21 (15)	8 (13)	13 (18)	0.483	–
Season	Summer	68 (50)	33 (52)	35 (47)	0.609	–
	Winter	69 (50)	30 (48)	39 (53)		
**APACHE II score (on admission)**		**18 (6)**	**14 (4)**	**21 (5)**	** < 0.001**	–
Mechanically ventilated at admission		115 (84)	56 (89)	59 (80)	0.167	–
**[SOFA minus renal] score (Day 0)**		**10 [7, 12]**	**8 [7, 10]**	**11 [9, 13]**	**< 0.001**	6.6
CRP on day 0 [mg/dL]		123 [54, 253]	98 [57, 187]	149 [54, 275]	0.057	11.7
Ionized calcium (Day 0) [mmol/L]		1.1 [1.1,1.2]	1.1 [1.1,1.2]	1.1 [1.1,1.2]	0.856	6.6
Received ECMO during stay in ICU		41 (30)	16 (25)	25 (34)	0.350	–
Plasma exchange during stay in ICU		3 (2)	1 (2)	2 (3)	1.000	–
**Kidney function at Day 5**	**AKI**	**51 (37)**	**0 (0)**	**51 (69**)	<**0.001**	**-**
	Recovered from AKI	23 (17)	0 (0)	23 (31)		
	Normal throughout	52 (38)	52 (83)	0 (0)		
	New AKI on day 5	11 (8)	11 (17)	0 (0)		
**ICU mortality**		**25 (18)**	**5 (8)**	**20 (27)**	**0.004**	–
**Hospital mortality**		**31 (23)**	**6 (10)**	**25 (34)**	**0.001**	–
	Missing data	2 (1)	1 (2)	1 (1)		
**Died within 30 days of enrolment**		**31 (23)**	**6 (10)**	**25 (34)**	**< 0.001**	–
	Missing data	7 (5)	6 (10)	1 (1)		
Hospital length of stay		22 [13, 35]	18 [13, 31]	28 [16, 45]	0.075	51.8

*AKI* Acute kidney injury, *APACHE* Acute physiology and chronic health evaluation, *CKD* Chronic kidney disease, *CRP* C-reactive protein, *ECMO* Extracorporeal membrane oxygenation, *GI* Gastrointestinal, *ICU* Intensive care unit, *SOFA* Sequential Organ Failure Assessment

Statistically significant variables are indicated in **bold**

Results are reported as number (%), mean (%) or median [interquartile range]

**Table 2:** Baseline characteristics based on kidney function and trajectories (as described in Fig. 1)

Variable	Category	All	Kidney Function at day 5					
			Normal throughout	Developed new AKI	AKI II/III throughout	Renal recovery from AKI II/III	*p*-value	Missing %
n		137	52	11	51	23		
Age		54 [47, 68]	50 [41, 69]	52 [51, 67]	57 [48, 66]	54 [48, 68]	0.776	0.7
Gender	Male	91 (66)	34 (65)	6 (55)	38 (75)	13 (57)	0.340	–
	Female	46 (34)	18 (35)	5 (45)	13 (25)	10 (43)		
Ethnicity	Asian	4 (3)	3 (6)	0	1 (2)	0	0.868	3.6
	Black	9 (7)	2 (4)	0	5 (10)	2 (9)		
	Caucasian	118 (86)	43 (83)	11 (100)	43 (84)	21 (91)		
	Other	1 (1)	1 (2)	0	0	0		
	Missing	5 (4)	3 (6)	0	2 (4)	0		
Season	Summer	68 (50)	27 (52)	6 (55)	20 (39)	15 (65)	0.201	0.0
	Winter	69 (50)	25 (48)	5 (45)	31 (61)	8 (35)		
**CRP (Day 0) [mg/dL]**		**123 [54, 253]**	**97 [53, 144]**	**117 [84, 300]**	**161 [68, 305]**	**110 [43, 218]**	**0.037**	**11.7**
Serum 25(OH)D on day 0 [nmol/L]		18 [10, 31]	20 [12, 34]	32 [14, 40]	14 [9, 26]	19 [9, 35]	0.076	6.6
Serum 1,25(OH)_2_D on day 0 [pmol/L]		50 [28, 68]	54 [33, 69]	63 [36, 106]	41 [26, 58]	36 [26, 56]	0.069	5.1
Calcium (Day 0) [mmol/L]		1.1 [1.1,1.2]	1.1 [1.1,1.2]	1.1 [1.1,1.2]	1.1 [1.1,1.2]	1.1 [1.1,1.2]	0.833	6.6
**APACHE II score (on admission)**		**18 [14, 21]**	**14 [12, 17**]	**15 [12, 16]**	**21 [19, 23]**	**20 [17, 24]**	** < 0.001**	**0.0**
**SOFA minus renal score (Day 0)**		**10 [7, 12]**	**8 [7, 10]**	**9 [6, 9]**	**11 [9, 13]**	**10 [8, 12]**	** < 0.001**	**6.6**
**ICU mortality**		25 (18)	4 (7.7)	1 (9)	18 (35.3)	2 (8.7)	**0.002**	**-**
**Hospital mortality**		31 (23)	5 (9.6)	1 (9)	22 (43.1)	3 (13)	** < 0.001**	**1.5**
**30-day mortality**		31 (23)	5 (9.6)	1 (9)	23 (45.1)	2 (8.7)	**0.001**	**5**
**Length of stay in ICU**		14 [8, 23]]	10 [5, 15]	16 [10, 19]	18 [10, 30]	18 [8, 27]	**0.010**	**29**

*AKI* Acute kidney injury, *APACHE* Acute physiology and chronic health evaluation, *CRP* C-reactive protein, *ICU* Intensive Care Unit, *SOFA* Sequential Organ Failure Assessment

Significant variables are indicated in **bold**

Results are reported as number (%), mean (%) or median [interquartile range]

### 25(OH) vitamin D results

**Fig. 2:**
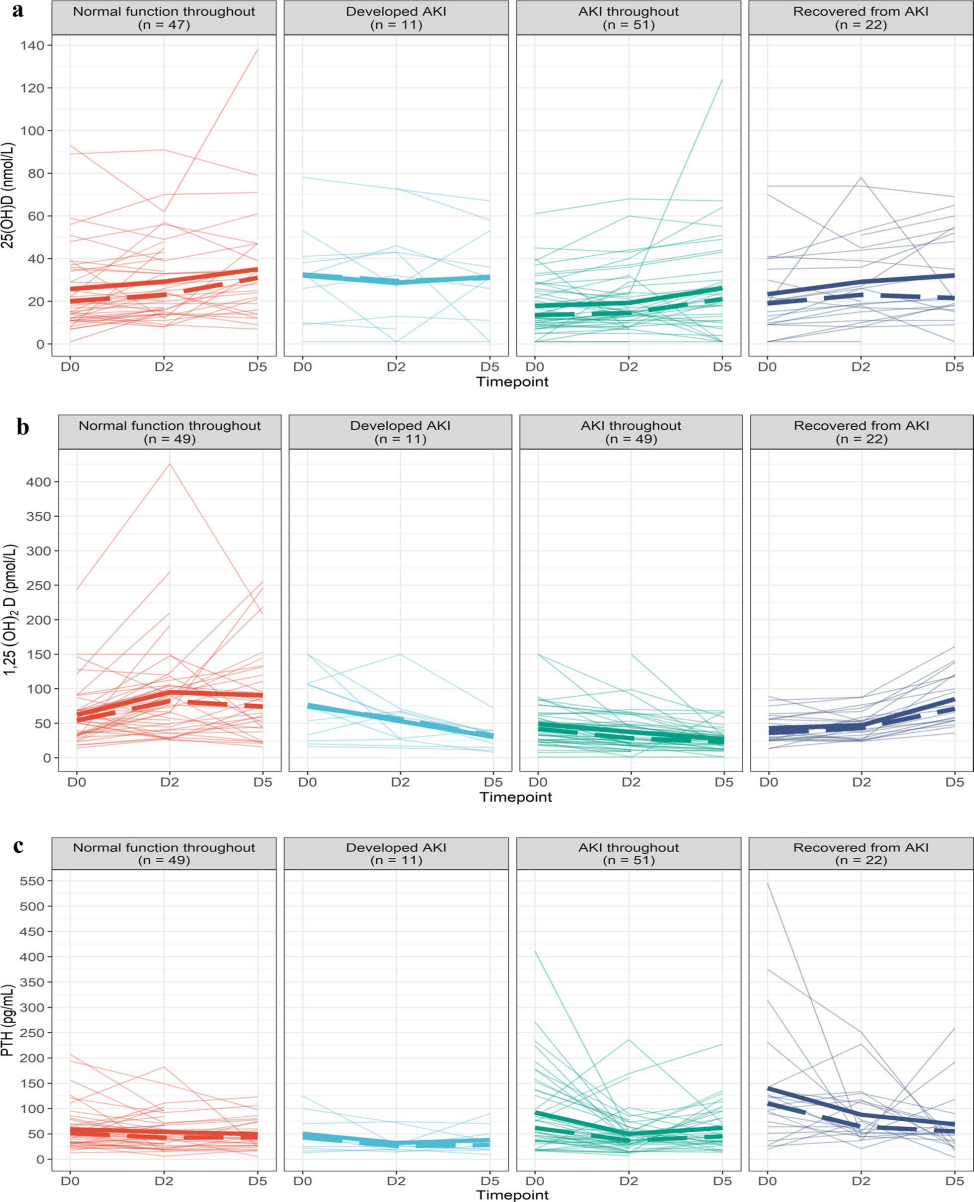
Changes in serum 25(OH)D, serum 1,25(OH)_2_D and plasma PTH concentrations of each patient by kidney function. **a** Serum 25(OH)D concentrations (*n* = 131). *AKI* Acute kidney injury **b** Serum 1, 25(OH)_2_D concentrations (*n* = 131). *AKI* Acute kidney injury. **c** Plasma PTH concentrations (*n* = 133). *AKI* Acute kidney injury, *PTH* Parathyroid hormone. Mean (solid) and median (dashed) results are highlighted at each time point. Faint lines represent individual patients.

Six patients had missing 25(OH)D data and were not included in the mixed model. Among the remaining 131 patients, serum 25(OH)D concentrations were low (median 18 nmol/L) on the day of enrolment across the whole patient cohort when compared with reference ranges for the general population. (Additional file 1: Table S1). In patients with AKI II/III at enrolment, serum 25(OH)D concentrations were approximately 5 nmol/l lower than in patients without AKI throughout the study, whether they recovered renal function or not. (Additional file 1: Table S2) In the three kidney function categories ‘normal kidney function throughout’, ‘AKI II/III throughout’, and ‘recovered from AKI II/III’, serum 25(OH)D levels increased between day 0 and day 5, but the changes were statistically not significant (Fig. 2a, Additional file 1: Tables S1 and Additional file 1: Figure S1).

Patients admitted in winter had significantly lower serum 25(OH)D concentrations than those admitted in summer. The mean difference was 12.0 nmol/l (95% CI 5.6 to 18.4; *p* < 0.001). (Additional file 1: Table S2) Among patients who developed new AKI and patients with AKI II/III who recovered kidney function during the 5-day period, females had a higher 25(OH)D result on the day of enrolment than males. (Additional file 1: Figure S2).

### Sensitivity analysis

The coefficients in the log-linear model had the same directions of association as the linear model. Male gender and winter season were independently associated with lower 25(OH)D results (Additional file 1: Table S3).

### 1,25(OH)_2_D results

Six patients (4%) had missing 1,25(OH)_2_D results and were not included in the mixed model. Serum 1,25(OH)_2_D concentrations rose between day 0 and day 5 in patients without AKI and in AKI patients who recovered kidney function during the 5-day follow-up period. (Fig. 2b and Additional file 1: Table S4) In contrast, in patients who did not have AKI at enrolment but developed new AKI, and in patients who had AKI II/III throughout the 5-day period, serum 1,25(OH)_2_D concentrations decreased during the follow-up period. Compared to the cohort with normal kidney function throughout, patients who developed new AKI and patients with AKI II/III throughout the 5-day period had significantly lower 1,25(OH)_2_D results at each time point. (Additional file 1: Tables S5 and S6) Similarly, AKI patients with renal recovery had significantly lower 1,25(OH)_2_D results at each time point compared to patients with normal kidney function throughout the 5-day period.

There was no difference in these trajectories within each category between male and female patients. (Additional file 1: figure S3) Patients admitted in winter had lower 1,25(OH)_2_D results than those admitted in summer with a mean difference of 9.8 pmol/l (95% CI − 0.8 to 20.4) which was statistically not significant (*p* = 0.069). (Additional file 1: Table S5).

### Sensitivity analysis

Patients with normal kidney function throughout and patients with AKI II/III who recovered kidney function during the 5-day period had higher 1,25(OH)_2_D levels than patients with persistent or new AKI. (Additional file 1: Table S6) Considering the variables outside the kidney function and time interaction, the log-linear model coefficients had the same directions of association as the linear model. (Additional file 1: Table S5 and S7) Age, gender, ethnicity, season, baseline CRP and baseline SOFA-renal score were not independently associated with 1,25(OH)_2_D concentrations.

### PTH

On day of enrolment, patients without AKI had lower plasma PTH concentrations compared to patients with AKI. (Fig. 2c and Additional file 1: Table S8) In all kidney function categories, the PTH results on day 5 were lower than on day 0 but the decline was most marked in patients with AKI who recovered kidney function during the 5-day period. (Additional file 1: Table S8 and Figure S3).

PTH concentrations were related to serum ionized calcium and magnesium concentrations. A serum ionized calcium concentration of 0.1 mmol/L above a patient’s average value was significantly (*p* < 0.001) associated with a PTH value 13.8 pg/ml lower (95% CI 7.4 to 20.1) than their average. (Additional file 1: Table S9) A magnesium value 0.1 mmol/l above a patient’s average was significantly (*p* = 0.040) associated with a PTH value 2.1 pg/ml higher (95% CI 0.1 to 4.2) than the patient’s average.

## Discussion

The key findings of this prospective two-centre observational study are: 1) Critically ill adult patients with AKI II/III had significantly lower serum 1,25(OH)_2_D concentrations compared to similarly unwell patients without AKI; 2) Serum 25(OH)D concentrations were low with no significant difference between critically ill patients with and without AKI; iii) In patients with AKI II/III, recovery of kidney function was associated with an increase in 1,25(OH)_2_D levels in contrast to patients with AKI II/III who did not recover kidney function; and iv) PTH concentrations were impacted by serum calcium and magnesium levels but did not correlate with changes in serum 1,25(OH)_2_D concentrations.

Vitamin D has pleiotropic effects on immunity, endothelial and mucosal functions, and glucose and calcium metabolism. 1,25(OH)_2_D is the main bioactive metabolite of vitamin D with the greatest physiological effect. Its half-life is much shorter (of the order of 4 h) than that of vitamin D3 (24 h) or 25(OH)D (3 weeks). Although deficiency is associated with poor outcomes [2, 17–19], the thresholds for sufficiency / deficiency often relate to bone health, without consideration of other physiological effects. Recommendations when to supplement Vitamin D differ in many countries. [18] The European Society for Clinical Nutrition and Metabolism considers a serum/plasma 25(OH)D concentration below 50 nmol/L (or 20 ng/ml) to define vitamin D deficiency [1]. A cut-off < 25 nmol/L (or 10 ng/mL) increases the risk for osteomalacia and nutritional rickets dramatically and is considered to determine severe vitamin D deficiency.

The optimal vitamin D concentration during critical illness is unknown. Besides critical illness itself, immobilization, fluid overload, plasma exchange and cardiopulmonary bypass have been reported to impact serum vitamin D concentrations. [2] Thus, the question remains whether low vitamin D values in critically ill patients reflect deficiency, greater severity of illness, the result of therapeutic interventions or are simply considered risk factors for poor outcomes. [18, 20] The role of routine Vitamin D supplementation in all critically ill patients remains under investigation but has not been explored specifically in critically ill patients with AKI. [9, 11, 18, 21]

Importantly, the relationship between AKI and vitamin D appears bi-directional. Loss of functioning nephrons impacts the 1-hydroxylase negatively and affects the ability to generate 1,25(OH)_2_D. [22, 23] On the other hand, experimental models have shown that vitamin D depletion contributes to AKI development due to several mechanisms, including upregulation of the renin–angiotensin–aldosterone system, increased expression of renal vascular renin and dysregulation of the immune system. [23–25] Vitamin D deficiency may also exacerbate AKI by worsening the renal vascular condition, preventing healing of renal ischaemia–reperfusion injury (IRI) and accelerating AKI-to-CKD progression. [25–28]

The role of supplementation with active vitamin D (calcitriol) in AKI has been investigated in experimental studies in rats, using different analogues in varying doses with heterogeneous results. Ersan et al. reported that pre-treatment with paricalcitol resulted in amelioration of IRI-induced AKI via a matrix metalloproteinase-dependent inflammatory mechanism. [29] Others showed that paricalcitol ameliorated liposaccharide (LPS)-associated AKI and cisplatin-induced AKI. [30, 31] Hamzawy et al. demonstrated that pre-treatment with oxacalcitriol ameliorated IRI-induced AKI through anti-inflammatory mechanisms. [32] In another experimental study, the administration of 1,25(OH)_2_D did not ameliorate gentamicin-induced AKI. [33] Arfian et al. investigated the effect of intraperitoneal administration of colecalciferol on IRI-induced AKI and found that AKI was mitigated through reduction in inflammation. [34] Outside AKI, two different oral doses of vitamin D (500 IU/kg and 1000 IU/kg) were used in an experimental model of paracetamol-induced liver failure. [35] The lower dose of 500 IU/kg was found to have a greater protective effect.

Our findings complement and advance the existing literature on Vitamin D metabolism in critically ill patients. Similar to previous reports, we found no difference in serum 25(OH)D concentrations between the AKI and non-AKI cohort. [7, 16] Instead, we demonstrated significant differences in 1,25(OH)_2_D concentrations between patients with and without severe AKI. By consensus, serum/plasma concentration of 25(OH)D is recognized as a valid biomarker for vitamin D status. [1] Our data suggest that 1,25(OH)_2_D deficiency exists and may be under-recognized, especially in critically ill patients with moderate to severe AKI. Importantly, colecalciferol has been used for supplementation in several clinical intervention studies but may be inappropriate and ineffective in patients with AKI. [36–38]

Our data shows that on day of enrolment, patients without AKI had lower plasma PTH concentrations compared to patients with AKI. In those who recovered kidney function by day 5, PTH levels declined again. These data are consistent with other human studies in the literature confirming acute elevations of PTH with AKI that subsequently returned to normal when AKI resolved. with resolution of AKI.. [39, 40] It is postulated that low concentrations of serum 1,25(OH)_2_D and secondary hypocalcaemia may contribute to secondary hyperparathyroidism in AKI.

It is well-known that after the induction of sustained hypocalcaemia, preformed PTH is secreted into the blood within 1 min and restoration of normocalcaemia results in a decrease in PTH levels with an apparent half-life of approximately 3 min. [41, 42]

Despite the strengths of our study, we acknowledge some limitations. First, we aimed to exclude patients taking vitamin D supplements from participating but acknowledge that it is possible that patients taking over-the-counter or prescribed vitamin D supplements may have been inadvertently included despite our best effort. The low levels of 25(OH)D observed and low variability within the data at day 0 suggest that this was not widespread. Second, we recruited patients in two tertiary care centres in the United Kingdom and acknowledge that our results may not be generalizable to patients in other geographical areas. Third, we included patients with AKI of any aetiology were included, promoting the generalizability of the findings. Nonetheless, we acknowledge that certain AKI sub-phenotypes may have a higher risk of 1,25(OH)_2_D deficiency than others. Consideration of AKI aetiology may be useful in further defining a population with the lowest vitamin D levels and to enrich future intervention studies. Fourth, we have some missing results because blood sampling was not done due to competing clinical requirements, or blood samples were underfilled and unsuitable for analysis. The statistical analyses were adjusted accordingly. Fifth, we did not measure FGF-23 and vitamin D binding protein consistently and reliably in all patients, as originally planned. We are aware that elevated serum FGF-23 correlate with increased mortality in critically ill patients independent of vitamin D status. Also, FGF-23 measurements may be outside the detectable, validated range for commercially available tests. [43] Sixth, we had planned to take samples on day of ICU discharge in all ICU survivors. However, this proved to be challenging. Our interim analysis after 50 patients showed that these samples had not been taken in more than 50% of patients due to a variety of reasons including emergency transfer to another hospitals or discharge from ICU outside usual working hours, patient refusal to have blood test taken, or simple human oversight. Further, it became apparent that length of stay in ICU after enrolment and day of ICU discharge were very variable between patients. For these reasons, we elected not to analyse the results to avoid misinterpretation. Seventh, we defined renal recovery as a reduction in AKI stage or return to baseline kidney function but know that serum creatinine is not a reliable biomarker of renal recovery. Further, we only monitored renal function for 5 days and acknowledge that renal recovery may take longer to develop. Finally, the majority of patients with AKI II/III received RRT during the 5-day study period and the sample size did not allow formal comparison between the RRT and non-RRT cohorts, as originally planned. However, in a previous study, we showed that vitamin D is not removed during RRT and that serum vitamin D concentrations did not significantly differ between patients with AKI III treated with or without RRT. [44]

## Conclusions

Among critically ill adult patients, serial serum concentrations of 1,25(OH)_2_D were significantly lower in patients with moderate-to-severe AKI compared to critically ill patients without AKI. There was no statistically significant difference in serum 25(OH)D concentrations. More research is needed to define vitamin D deficiency in critically ill patients with AKI. Our results support a case to investigate the health benefits and safety of supplementation with active vitamin D analogues in this cohort.

### Supplementary Information

**Additional file 1. Figure S1:** Changes in 25(OH)D measurements by kidney function and gender. **Table S1a:** Serum 25(OH)D concentrations by kidney function and time point. **Table S1b:** Coefficient estimates for the final 25(OH)D model.

## Data Availability

The datasets used and/or analysed during the current study are available from the corresponding author on reasonable request.

## Abbreviations

AKI: Acute kidney injury
AKD: Acute kidney disease
AOR: Adjusted odds ratio
APACHE: Acute physiology and chronic health evaluation
CI: Confidence interval
CKD: Chronic kidney disease
CRP: C-reactive protein
CV: Coefficient of variation
ECMO: Extracorporeal membrane oxygenation
eGFR: Estimated glomerular filtration rate
ESKD: End stage kidney disease
FGF-23: Fibroblast growth factor-23
GFR: Glomerular filtration rate
GI: Gastrointestinal
ICU: Intensive care unit
IQR: Interquartile range
IRI: Ischaemia-reperfusion injury
KDIGO: Kidney Disease Improving Global Outcomes
LPS: Liposaccharide
PTH: Parathyroid hormone
RRT: Renal replacement therapy
SOFA: Sequential Organ Failure Assessment
TPN: Total parenteral nutrition
UVB: Ultraviolet B
