# Supplementary material for: Vitamin D metabolism in critically ill patients with acute kidney injury: a prospective observational study
Source: Crit Care. 2024 Apr 2;28:108. doi: 10.1186/s13054-024-04869-4 (PMC10988948; doi:10.1186/s13054-024-04869-4)
Supplement: Supplementary file 1 — Additional file 1. Figure S1: Changes in 25(OH)D measurements by kidney function and gender. Table S1a: Serum 25(OH)D concentrations by kidney function and time point. Table S1b: Coefficient estimates for the final 25(OH)D model. [file 13054_2024_4869_MOESM1_ESM.docx]

**Supplementary Table S1: Serum 25(OH)D concentrations by kidney function and time point**

**Median values [IQR]** [nmol/L]

| **Kidney function during 5-day follow up period** | **Day 0** | **Day 2** | **Day 5** | **Overall** |
| --- | --- | --- | --- | --- |
| **Normal kidney function** | 20 [12,34] | 23 [14,40] | 31 [19,39] | 22 [14,37] |
| **Developed new AKI** | 32 [14,40] | 30 [8,43] | 31 [15,49] | 32 [10,43] |
| **AKI II/III throughout** | 14 [9,26] | 14 [10,24] | 21 [11,31] | 16 [9,28] |
| **Recovered from AKI II/III** | 19 [9,35] | 23 [14,40] | 22 [18,52] | 20 [11,40] |
| **Overall** | 18 [10,31] | 18 [12,36] | 25 [14,44] | 20 [11,35] |

Abbreviations: AKI = acute kidney injury; IQR = interquartile range

**Supplementary Table S2: Coefficient estimates for the final 25(OH)D model** (n = 277 observations from 107 patients)

| **Variable** | **Category** | **Estimate *** | **95% CI** | **P-value** |
| --- | --- | --- | --- | --- |
| Kidney function (ref: Normal throughout) | Developed AKI | 7.6 | -4.6 to 19.8 | 0.221 |
|  | AKI II/III throughout | -5.4 | -13.6 to 2.8 | 0.197 |
|  | Recovered from AKI II/III | -4.5 | -13.8 to 4.7 | 0.336 |
| **Time since recruitment (days)** |  | **1.1** | **0.4 to 1.8** | **0.002** |
| Age (years) |  | 0.1 | -0.1 to 0.3 | 0.266 |
| Gender (ref: Male) | Female | 4.4 | -2.4 to 11.2 | 0.204 |
| Ethnicity (ref: White) | Non-white | -7.3 | -17.0 to 2.4 | 0.138 |
| **Season (ref: Summer)** | **Winter** | **-12.0** | **-18.4 to -5.6** | **<0.001** |
| Baseline CRP |  | -0.01 | -0.04 to 0.01 | 0.315 |
| Baseline SOFA minus renal score |  | -0.4 | -1.5 to 0.7 | 0.441 |

Abbreviations: AKI = acute kidney injury; CI = confidence interval; CRP = C reactive protein; SOFA = Sequential Organ Failure Assessment

Significant variables are indicated in **bold**.

* Estimates are the ratio of means

**Supplementary Table S3: Coefficient estimates for the final log-linear 25(OH)D model**

| **Variable** | **Category** | **Estimate *** | **95% CI** | **P-value** |
| --- | --- | --- | --- | --- |
| Kidney function during 5-day period  (reference: normal kidney function throughout) | Developed new AKI | 1.15 | 0.64 to 2.07 | 0.642 |
|  | AKI II/III throughout | 0.71 | 0.48 to 1.06 | 0.096 |
|  | Recovered from AKI II/III | 0.72 | 0.46 to 1.13 | 0.146 |
| Time since recruitment (days) |  | 1.04 | 1.00 to 1.09 | 0.077 |
| Age (years) |  | 1.004 | 0.994 to 1.015 | 0.422 |
| **Gender** (reference: Male) | **Female** | **1.45** | **1.04 to 2.01** | **0.030** |
| Ethnicity (ref: White) | Non-white | 0.89 | 0.56 to 1.42 | 0.622 |
| **Season** (reference: Summer) | **Winter** | **0.68** | **0.50 to 0.93** | **0.016** |
| Baseline CRP |  | 0.999 | 0.998 to 1.001 | 0.321 |
| Baseline [SOFA-renal] score |  | 0.99 | 0.94 to 1.04 | 0.606 |

Abbreviations: AKI = acute kidney injury; CRP = C-reactive protein; SOFA = Sequential Organ Failure Assessment; CI = confidence interval

* Estimates are the ratio of means

Significant variables are indicated in **bold**.

**Supplementary Table S4 Serum 1,25(OH)_2_D concentrations by kidney function and time point**

**Median values [IQR]** [pmol/L]

| **Kidney function during 5-day follow up period** | **Day 0** | **Day 2** | **Day 5** | **Overall** |
| --- | --- | --- | --- | --- |
| **Normal kidney function** | 54 [33,69] | 82 [45,106] | 74 [44,123] | 65 [36,94] |
| **Developed new AKI** | 74 [29,107] | 56 [27,67] | 32 [16,38] | 37 [23,71] |
| **AKI II/III throughout** | 42 [26,61] | 28 [18,48] | 23 [13,33] | 32 [20,49] |
| **Recovered from AKI II/III** | 36 [26,56] | 43 [27,58] | 71 [54,118] | 53 [30,72] |
| **Overall** | 50 [28,68] | 46 [27,72] | 44 [22,76] | 47 [26,71] |

Abbreviations: AKI = acute kidney injury; IQR = interquartile range

**Supplementary Table S5: Coefficient estimates for the final log-linear 1,25(OH)_2_D model** (n = 288 observations from 108 patients)

| **Variable** | **Category** | **AOR** | **95% CI** | **P-value** |
| --- | --- | --- | --- | --- |
| Kidney function during 5-day period (reference: Normal kidney function throughout) | Developed new AKI | 0.99 | 0.58 to 1.70 | 0.980 |
|  | **AKI II/III throughout** | **0.54** | **0.37 to 0.78** | **0.001** |
|  | **Recovered from AKI II/III** | **0.57** | **0.39 to 0.82** | **0.003** |
| **Time since enrolment (days)** |  | **1.08** | **1.02 to 1.14** | **0.010** |
| **Kidney function X Time (ref: Normal X Time)** | **Developed new AKI X Time** | **0.78** | **0.70 to 0.87** | **<0.001** |
|  | **AKI II/III throughout X Time** | **0.81** | **0.75 to 0.88** | **<0.001** |
|  | Recovered from AKI II/III X Time | 1.06 | 0.99 to 1.13 | 0.096 |
| Age (years) |  | 1.002 | 0.994 to 1.011 | 0.615 |
| Gender (reference: Male) | Female | 1.22 | 0.93 to 1.60 | 0.143 |
| Ethnicity (reference: White) | Non-white | 1.11 | 0.75 to 1.63 | 0.599 |
| Season (reference: Summer) | Winter | 0.80 | 0.62 to 1.04 | 0.091 |
| Baseline CRP |  | 1.001 | 1.000 to 1.002 | 0.130 |
| Baseline SOFA minus Renal score |  | 1.04 | 1.00 to 1.09 | 0.055 |

Abbreviations: AKI = acute kidney injury; AOR = adjusted odds ratio; CRP = C-reactive protein; SOFA = Sequential Organ Failure Assessment; CI = confidence interval

Estimates are the ratio of means.

Significant variables are indicated in **bold**.**Supplementary Table S6: Kidney function time trends for the final log-linear 1,25(OH)_2_D model** (n = 288 observations from 108 patients)

| **Kidney function** | **Estimate** | **95% CI** | **P-value** |
| --- | --- | --- | --- |
| **Normal kidney function**  **Throughout** | **1.10** | **1.04 to 1.17** | **0.001** |
| **Developed new AKI** | **0.83** | **0.76 to 0.92** | **<0.001** |
| **AKI II/III throughout** | **0.87** | **0.82 to 0.91** | **<0.001** |
| **Recovered from AKI II/III** | **1.14** | **1.11 to 1.17** | **<0.001** |

Abbreviations: AKI = acute kidney injury; CI = confidence interval

Estimates are the ratio of means. Statistically significant variables are indicated in **bold**.

**Supplementary Table S7: Coefficient estimates for the final 1,25(OH)_2_D model** (n = 288 observations from 108 patients)

| **Variable** | **Category** | **Estimate** | **95% CI** | **P-value** |
| --- | --- | --- | --- | --- |
| KF (ref: Normal throughout) | Developed AKI | 4.9 | -29.1 to 38.9 | 0.774 |
|  | **AKI throughout** | **-29.3** | **-52.6 to -5.9** | **0.015** |
|  | **Recovered from AKI** | **-37.2** | **-63.0 to -11.3** | **0.005** |
| **Time since recruitment (days)** |  | **7.2** | **1.0 to 13.4** | **0.022** |
| **KF X Time (ref: Normal X Time)** | **Developed AKI X Time** | **-16.6** | **-25.6 to -7.5** | **<0.001** |
|  | **AKI throughout X Time** | **-12.3** | **-18.8 to -5.7** | **<0.001** |
|  | Recovered from AKI X Time | 0.7 | -6.4 to 7.7 | 0.854 |
| Age (years) |  | 0.04 | -0.3 to 0.4 | 0.844 |
| Gender (ref: Male) | Female | 6.5 | -5.0 to 18.1 | 0.265 |
| Ethnicity (ref: White) | Non-white | 7.2 | -9.4 to 23.7 | 0.391 |
| Season (ref: Summer) | Winter | -9.8 | -20.4 to 0.8 | 0.069 |
| Baseline CRP |  | 0.01 | -0.03 to 0.05 | 0.616 |
| **Baseline SOFA minus renal score** |  | **2.5** | **0.6 to 4.3** | **0.009** |

Abbreviations: AKI = acute kidney injury; AOR = adjusted odds ratio; CRP = C-reactive protein; SOFA = Sequential Organ Failure Assessment; CI = confidence interval

Estimates are the ratio of means.

Significant variables are indicated in **bold**.

**Supplementary Table S8: Median plasma PTH concentrations by kidney function and time point**

| **Kidney function during 5-day follow up period** | **Day 0** | **Day 2** | **Day 5** | **Overall** |
| --- | --- | --- | --- | --- |
| **Normal kidney function** | 51 [32,65] | 43 [30,69] | 43 [27,74] | 45 [30,69] |
| **Developed new AKI** | 50 [29,84] | 27 [19,54] | 33 [20,60] | 34 [20,70] |
| **AKI II/III throughout** | 60 [37,105] | 37 [26,56] | 45 [31,82] | 46 [30,80] |
| **Recovered from AKI II/III** | 110 [66,141] | 64 [48,115] | 55 [33,72] | 70 [46,120] |
| Overall | 57 [35,98] | 44 [27,72] | 44 [28,76] | 49 [30,81] |

Abbreviations: AKI = acute kidney injury; PTH = parathyroid hormone; IQR = interquartile range

Medians and IQR are shown

Results presented as: median [IQR] in pg/ml

**Supplementary Table S9: Coefficient estimates for PTH model** (n = 274 observations from 108 patients)

| **Variable** | **Category** | **Estimate** | **95% CI** | **P-value** |
| --- | --- | --- | --- | --- |
| 1,25(OH)_2_D (pmol/L), WP effect |  | 0.03 | -0.05 to 0.12 | 0.447 |
| 1,25(OH)_2_D (pmol/L), BP effect |  | 0.04 | -0.14 to 0.23 | 0.654 |
| Kidney function (ref: Normal throughout) | Developed new AKI | -20.9 | -43.7 to 1.8 | 0.070 |
|  | AKI II/III throughout | 4.6 | -15.6 to 24.9 | 0.650 |
|  | **Recovered from AKI II/III** | **45.2** | **13.4 to 77.0** | **0.006** |
| Time since recruitment (days) |  | -1.3 | -2.9 to 0.40 | 0.136 |
| Age (years) |  | 0.22 | -0.27 to 0.71 | 0.374 |
| Gender (ref: Male) | Female | -2.1 | -18.2 to 13.9 | 0.792 |
| Ethnicity (ref: White) | Non-white | -14.6 | -37.0 to 7.8 | 0.199 |
| Season (ref: Summer) | Winter | 1.1 | -13.3 to 15.4 | 0.885 |
| Baseline CRP |  | -0.02 | -0.08 to 0.03 | 0.410 |
| Baseline SOFA-renal score |  | 0.73 | -1.9 to 3.4 | 0.581 |
| **Ionised Calcium (mmol/L) WP effect** |  | **-137.5** | **-200.7 to -74.3** | **<0.001** |
| Ionised Calcium (mmol/L) BP effect |  | -77.5 | -228.7 to 73.7 | 0.311 |
| **Magnesium (mmol/L) WP effect** |  | **21.3** | **0.97 to 41.6** | **0.040** |
| Magnesium (mmol/L) BP effect |  | 10.8 | -40.8 to 62.4 | 0.678 |

Abbreviations: Abbreviations: AKI = acute kidney injury; CRP = C-reactive protein; SOFA = Sequential Organ Failure Assessment; CI = confidence interval; BP = Between-person; WP = Within-person;

Significant variables are indicated in **bold**.

**Supplementary figures**

**Supplementary Figure S1** **Changes in 25(OH)D measurements by kidney function and gender**


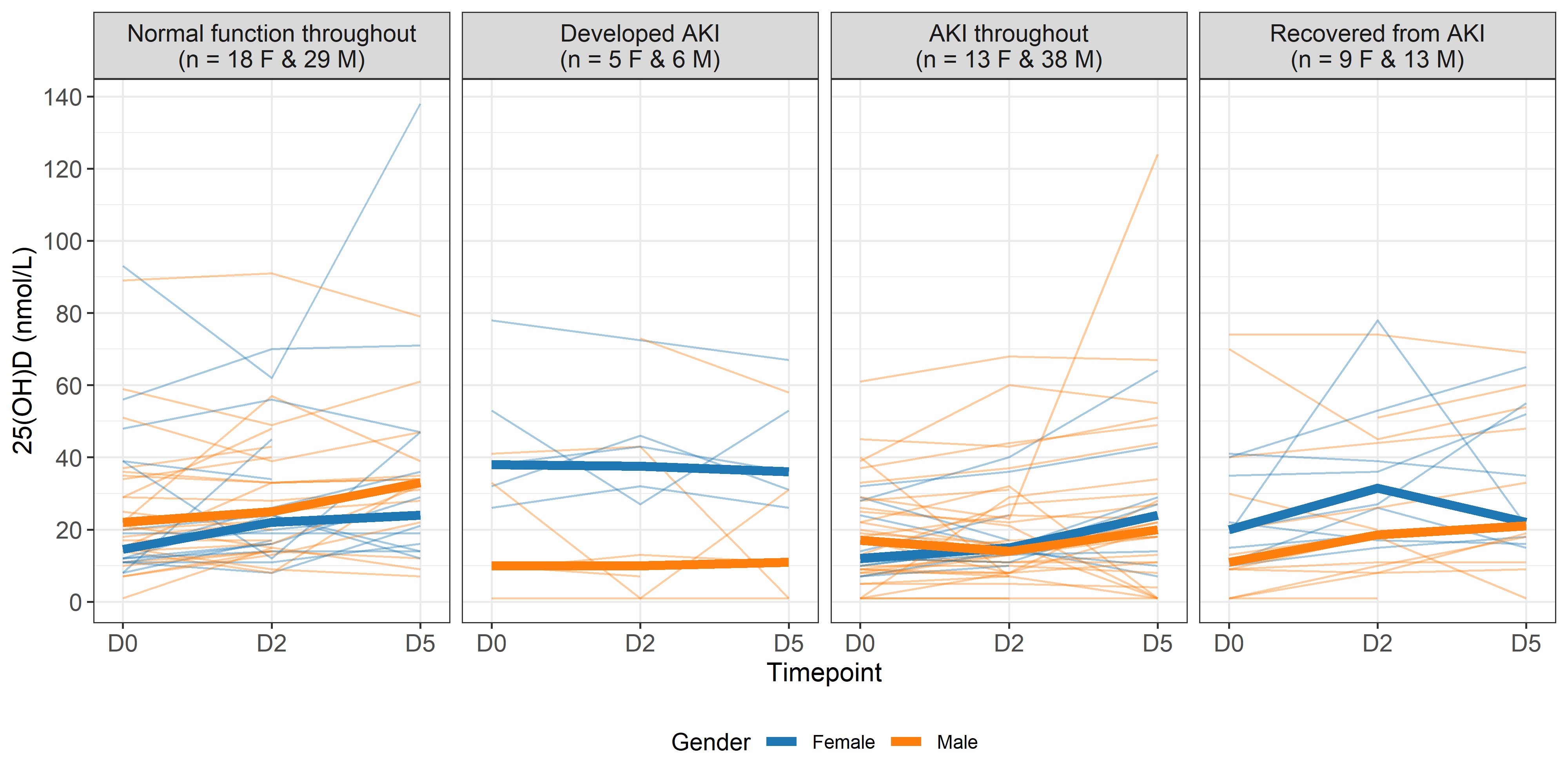


We highlighted the median measurement at each time point.

Faint lines represent individual patients

**Supplementary Figure S2: Changes in 1,25(OH)_2_D measurements for each patient by kidney function and gender**


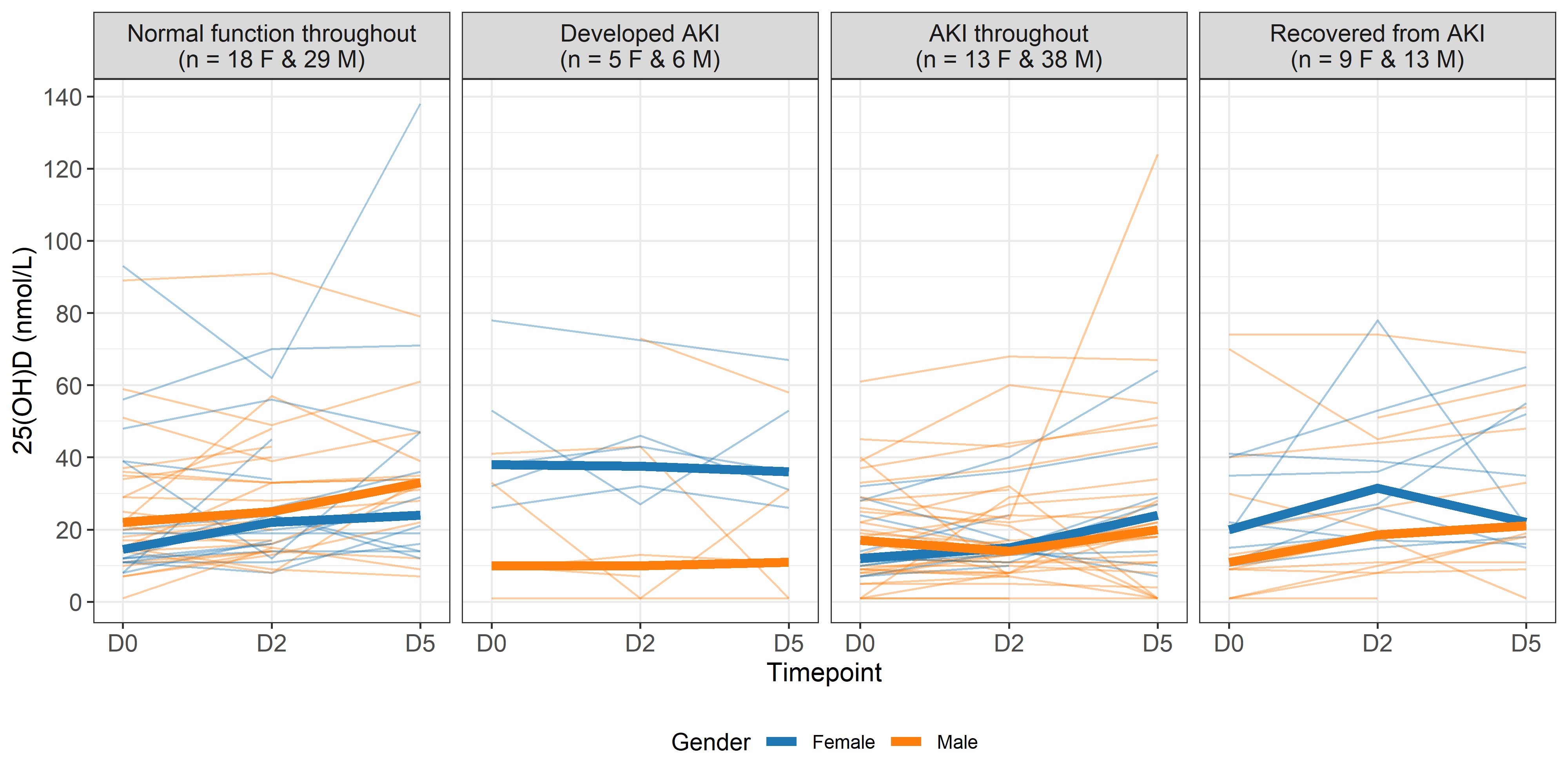


We highlighted the median measurement at each time point.

Faint lines represent individual patients

**Supplementary Figure S3: Changes in plasma PTH concentrations for each patient by kidney function and gender (n = 133)**


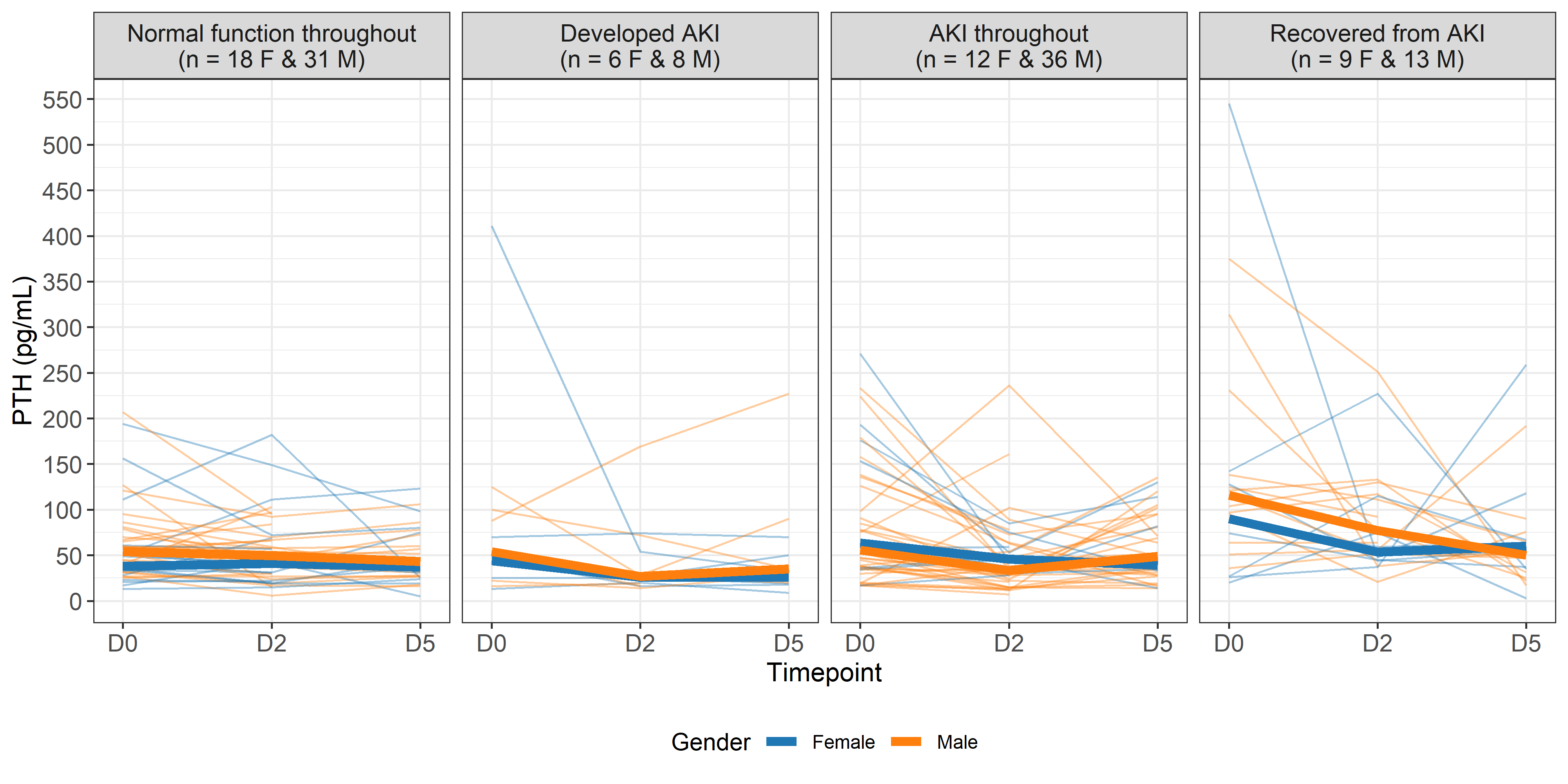


We highlighted the median measurement at each time point.

Faint lines represent individual patients
